# Thiamine administration may increase survival benefit in critically ill patients with myocardial infarction

**DOI:** 10.3389/fnut.2023.1227974

**Published:** 2023-08-29

**Authors:** Suru Yue, Jia Wang, Yumei Zhao, Enlin Ye, Dongdong Niu, Jiasheng Huang, Xiaolin Li, Yiling Hu, Xuefei Hou, Jiayuan Wu

**Affiliations:** ^1^Clinical Research Service Center, Affiliated Hospital of Guangdong Medical University, Zhanjiang, Guangdong, China; ^2^Guangdong Engineering Research Center of Collaborative Innovation of Clinical Medical Big Data Cloud Service in Western Guangdong Medical Union, Affiliated Hospital of Guangdong Medical University, Zhanjian, Guangdong, China

**Keywords:** myocardial infarction, cardiovascular disease, thiamine, critically ill patients, mortality risk, MIMIC-IV database

## Abstract

**Background:**

Myocardial infarction (MI) is a common cardiovascular disease (CVD) in critically ill patients, leading to 17% mortality in the intensive care unit (ICU) setting. Patients with CVD frequently suffer from thiamine insufficiency, thereby thiamine supplements may be helpful. Unfortunately, the relationship between thiamine treatment and survival outcomes in ICU patients with MI is still unknown. The purpose of the research is to demonstrate the survival advantage of thiamine application in these patients.

**Methods:**

The Medical Information Mart of Intensive Care-IV database served as the foundation for this retrospective cohort analysis. Depending on whether patients were given thiamine therapy during the hospital stay, critically ill MI patients were split into the thiamine and non-thiamine groups. The Kaplan–Meier (KM) method and Cox proportional hazard models were used to evaluate the relationship between thiamine use and the risk of in-hospital, 30-day, and 90-day mortality. To validate the results, a 1:2 closest propensity-score matching (PSM) was also carried out.

**Results:**

This study included 1782 patients for analysis with 170 and 1,612 individuals in the thiamine and non-thiamine groups, respectively. The KM survival analyses revealed that the risk of in-hospital, 30-day, and 90-day mortality was significantly lower in the thiamine group than the none-thiamine group. After modifying for a variety of confounding factors, the Cox regression models demonstrated substantial positive impacts of thiamine use on in-hospital, 30-d, and 90-d mortality risk among critically ill patients with MI with hazard ratio being 0.605 [95% confidence interval (CI): 0.397–0.921, *p* = 0.019], 0.618 (95% CI: 0.398–0.960, *p* = 0.032), and 0.626 (95% CI: 0.411–0.953, *p* = 0.028), respectively, in the completely modified model. PSM analyses also obtained consistent results.

**Conclusion:**

Thiamine supplementation is related to a decreased risk of mortality risk in critically ill patients with MI who are admitted to the ICU. More multicenter, large-sample, and well-designed randomized controlled trials are needed to validate this finding.

## Introduction

Myocardial infarction (MI) is defined as myocardial cell death due to prolonged ischaemia and is a major contributor of mortality in cardiovascular disease (CVD) (1). Given that critically ill patients are vulnerable to myocardial injury from various causes including ischemia and non-ischemia, MI is a common disease in intensive care units (ICUs) and coronary care units. Studies have shown that 66% of patients hospitalized for MI are admitted to the ICU on the first day of admission, and the ICU mortality rate is as high as 25.6% (2). Over the past decades, advances in pharmacology, catheter basis, and surgical reperfusion have made substantial progress in improving the outcomes of critically ill patients with MI. In particular, the incidence of mechanical complications in critically ill patients with MI significantly decreased after the introduction of percutaneous coronary intervention. However, patients with large infarcts or those who do not receive timely revascularization remain at risk for mechanical complications. These complications significantly increase morbidity, mortality, and hospital resource utilization, resulting in up to 60% mortality even with appropriate treatment (3, 4). Moreover, the economic burden caused by MI should not be ignored. The American Heart Association estimates that the direct and indirect costs of CVD in the USA will increase from $272.5 billion and $171.7 billion in 2010 to $818.1 billion and $275.8 billion in 2030, respectively (5). In particular, with the introduction of active treatment such as reperfusion therapy, the overall mortality of MI decreases by 40% and the incidence of in-hospital complications is also substantially reduced (5, 6). Hunziker et al. reported that over the past 20 years, with the implementation of reperfusion therapy, the total mortality of MI patients has decreased from 8.7 to 7.3%, and the incidence of in-hospital cardiogenic shock has decreased from 7.8 to 3.5% with its corresponding in-hospital mortality declining from 62.2 to 36.3% (7). Therefore, finding new therapeutic and cheaper interventions is needed to improve survival outcomes and reduce disease burden in critically ill patients with MI.

Micronutrient deficiency may reduce energy generation in cardiomyocytes and lead to poor clinical outcomes in patients with CVD (8). Thiamine, also known as vitamin B1, is an essential water-soluble vitamin that cannot be synthesized by the human body. In the human body, thiamine has three forms, namely, thiamine monophosphate, thiamine pyrophosphate (TPP), and thiamine triphosphate. TPP is the main bio-active form of thiamine and one of the best markers of the overall nutritional status. Moreover, TPP is a co-factor in the pyruvate and 2-hydroxyvallutarate dehydrogenase complex and is an indispensable coenzyme involved in mitochondrial oxidative decarboxylation, playing an essential role in the synthesis of adenosine triphosphate (ATP) in mitochondria (9). Thiamine also maintains the cellular redox state by participating in the pentose–phosphate cycle for NADPH and glutathione synthesis (10). Recently, thiamine derivatives have been found to have the nonenzymatic functions involved in gene expression, stress response, and regulation of neural signal transduction (10, 11). When thiamine is deficient, the activity of pyruvate dehydrogenase complex, transketolase, and α-ketoglutarate dehydrogenase are reduced, resulting in low ATP synthesis, limited supply and circulation of Krebs cycle, and cell oxidative damage and death (12). Thiamine is primarily transported to organs and tissues with high metabolic requirements and affects high metabolic systems, such as heart, brain, muscle, and nerves (12). Absolute or relative thiamine depletion is reportedly associated with a nearly 50% increase in patient mortality when occurring in adult and pediatric patients with critical illness (13). In conclusion, giving thiamine supplements to individuals who are critically ill can improve their prognosis.

Thiamine supplementation has been studied among severely ill patients. A double-blind randomized controlled trial in patients with septic shock has shown that thiamine supplementation could significantly reduce serum lactate levels for over 24 h and reduce mortality in patients with baseline thiamine deficiency (14). Woolum et al. also found that thiamine treatment could increase the lactate clearance rate and decrease the 28-day mortality in patients with septic shock (15). Other studies have demonstrated that thiamine therapy (also known as HAT therapy), which unites thiamine with hydrocortisone and ascorbic acid, could reverse shock organ dysfunction and decrease mortality in critically ill patients. For example, Iglesias et al. evaluated metabolic resuscitation in sepsis patients and found that the time of shock response of patients who have received HAT treatment is significantly lower than that of patients not treated with HAT therapy (16). Severe pneumonia patients treated with HAT also have a significantly lower in-hospital mortality than those not treated with HAT in the ICU (17). Thiamine deficiency in the body tends to cause lactate accumulation, reducing peripheral resistance, and thereby increasing cardiac preload. Increased cardiac preload combined with myocardial injury and dysfunction may be an etiological basis of cardiovascular events when thiamine deficiency occurs in critically ill patients. Existing evidence suggests that intravenous thiamine may help correct lactic acidosis in critically ill patients, thereby improving heart function and reducing mortality (18). However, no evidence shows whether thiamine supplementation helps improve the outcomes of critically ill patients with MI. Given the convenience and low cost of thiamine administration, it may be an effective approach to treating critically ill patients with MI. Therefore, in order to improve patient treatment and offer evidence for clinical decision-making, the current study intends to explore the impact of thiamine supplementation on the prognosis of MI patients using the Medical Information Mart for Intensive Care (MIMIC)-IV database.

## Methods

### Data foundation

This study was a single-center retrospective observational study. Data of critically ill patients with MI were extracted from the MIMIC-IV (version 2.2) database. MIMIC-IV, an updated version of MIMIC-III released on January 6, 2023, is an online accessible clinical critical care database containing comprehensive data on over 200,000 patients between 2008 and 2019 in Beth Israel Deaconess Medical Center (19). The database was approved by the Institutional Review Boards of the Massachusetts Institute of Technology and the Beth Israel Deaconess Medical Center. MIMIC-IV used anonymized personal identifier to protect the privacy of all patients, so informed consent was not required. To obtain access, the authors finished the correlative lessons and got the corresponding certificate (no. 9983480).

### Participants

The International Classification of Diseases 9 and 10 codes were used to diagnosis MI in all cases. Only the information from the patient’s initial admission was chosen if they were admitted to the ICU more than once. Patients suffering fewer than 48 h of hospital or ICU stay, more than 20% missing information, patients under the age of 18, or patients with diseases unsuitable for thiamine therapy were disqualified. Depending on whether or not they had received thiamine treatment during hospitalization (including *via* intravenous and oral methods), MI patients were split into the thiamine and no-thiamine groups.

### Data collection

After determining the stay identity of the selected patients, data extraction was performed using the PostgreSQL tool (v.14, PostgreSQL Global Development Group, Berkeley, CA, United States). The following characteristics were extracted: (1) demographic information, containing age, sex, ethnicity, and body mass index (BMI); (2) clinical scores, involving the Glasgow Coma Scale (GCS) and the sequential organ failure assessment (SOFA) score; (3) complications, containing congestive heart failure, diabetes, chronic renal disease, cerebrovascular disease, chronic pulmonary disease, and sepsis; (4) laboratory parameters, containing glucose, hemoglobin, sodium, lactate, blood urea nitrogen (BUN), platelets, creatinine, white blood cell, calcium, hydrogen ion concentration (pH), potassium, prothrombin time, and partial prothrombin time; (5) vital signs, containing heart rate, respiratory rate, body temperature, oxygen saturation (SpO2), systolic blood pressures, diastolic blood pressures, mean blood pressures, and urine output; and (6) clinical therapy, containing vasopressor, renal replacement therapy (RRT), and mechanical ventilation. The first measuring parameter utilized in this investigation were taken 24 h after ICU admission. The primary outcome of this study was in-hospital. Secondary outcomes included 30-day and 90-day mortality. No attempt was made to assess the study’s sample size because it was an epidemiological study with a hypothesis. To get the greatest statistical power, all eligible patients in the MIMIC-IV database were enrolled.

### Statistical analysis

Normality tests showed that all continuous variables had no normal distribution in this study, so they were shown as medians and quartiles (Supplementary Table S1). The Mann–Whitney U test was used to compare the differences between the two groups. Categorical variables were displayed as numbers and percentages, and the chi-square test or Fisher’s exact test was used to identify between-group differences. To determine if thiamine supplementation had an impact on the survival results, Kaplan–Meier (KM) curves and the log-rank test were used.

Cox regression models with hazard ratios (HRs) and 95% confidence intervals (CIs) were built to evaluate the impact of thiamine administration on prognosis by controlling various confounding factors. The crude model only included whether thiamine was used without adjustment of any covariate. In model 1, we adjusted for demographic characteristics. In model 2, we further adjusted for comorbidities. In model 3, we additionally adjusted for clinical scores. In model 4, we further added vital signs into the Cox regression model. In model 5, covariates were additionally adjusted for laboratory tests. Finally, model 6 was additionally adjusted for clinical therapy. The proportional hazard (PH) assumption of the Cox regression model was assessed by the Schoenfeld residual method and deviance residual plot. Multicollinearity between independent variables was tested using variance inflation factors (VIFs) before multivariate Cox regression. This study minimized the baseline disparities between the two groups using a 1:2 closest propensity score matching (PSM) to assure stability. In observational studies, we are unable to achieve the randomization grouping, but PSM can be used to eliminate the imbalance of confounding factors (20). PSM can match the observation and control groups in accordance with the propensity score and then remove the participants who do not match, making sure that the matched participants are comparable in potential confounding factors with the exception of exposure factors (20, 21). Therefore, when there is a difference between the outcomes of the observation group and the control group after matching, we can attribute the difference to the exposure factors. Multiple imputation method was applied with the “mice” package of R software to fill in the missing data based on the random forest method repeated 500 times (22). MI can create multiple datasets with different insert values and perform statistical analysis, thus merging the final results to give a valid synergistic estimate. To validate the robustness and reliability of the results, sensitivity analyses were performed by excluding individuals with missing data. Moreover, subgroup analyses were conducted that took into account age, gender, ethnicity, BMI, and complications. Statistical significance was defined as a two-tailed probability value of *p* < 0.05. R software (version 4.1.0) was used to conduct all of the analyses.

## Results

### Baseline features

**Figure 1:**
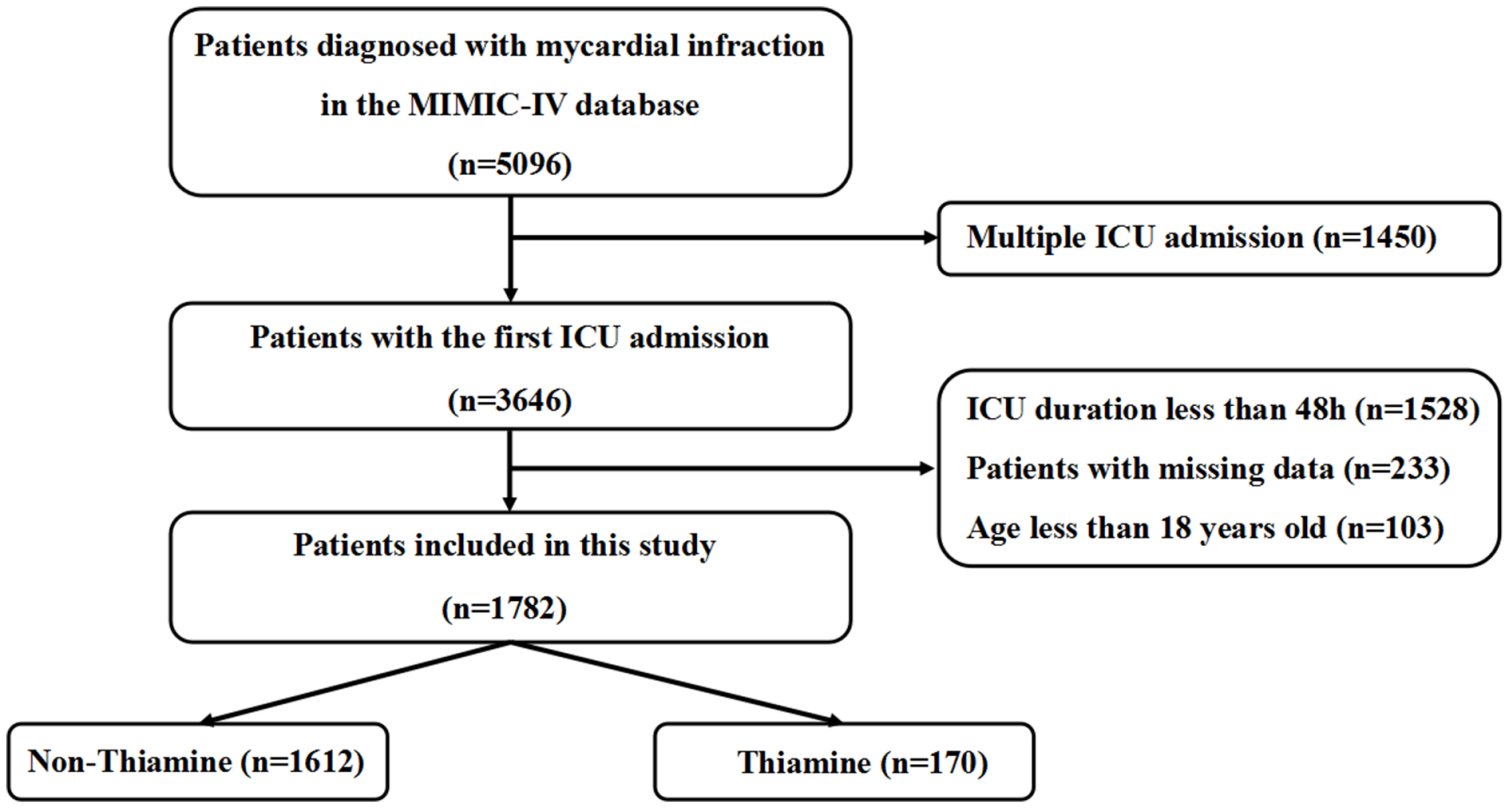
Inclusion and exclusion flowchart of the study.

A total of 5,096 patients diagnosed with MI were originally taken from the MIMIC-IV database in ICU. Based on the exclusion criteria, 3,314 patients were excluded (Figure 1). Ultimately, 1,782 patients were included, comprising 170 (9.5%) patients who received thiamine therapy during hospitalization. Table 1 displays the variations in baseline features between the thiamine and non-thiamine groups.

**Table 1:** Baseline features of the original and PSM populations.

Variable	Original population					PSM population					Missing ratio (%)
	Total (*n* = 1782)	None-Thiamine (*n* = 1,612)	Thiamine (n = 170)	*p* value	SMD	Total (*n* = 498)	None-Thiamine (*n* = 332)	Thiamine (*n* = 166)	*p* value	SMD	
Age, years	73.3 (64.3, 82.0)	73.9 (64.9, 82.6)	68.0 (57.4, 75.5)	< 0.001	0.529	67.3 (58.7, 76.8)	67.1 (58.8, 77.6)	68.1 (58.5, 76.2)	0.753	0.024	0.0
Gender, *n* (%)				< 0.001	0.323				0.864	0.016	0.0
Male	1,078 (60.5)	925 (59.1)	126 (74.1)			368 (73.9)	246 (74.1)	122 (73.5)			
Female	704 (39.5)	660 (40.9)	44 (25.9)			130 (26.1)	86 (25.9)	44 (26.5)			
BMI, kg/m^2^	27.5 (23.7, 31.8)	27.5 (23.7, 31.8)	27.7 (23.7, 31.6)	0.785	0.021	27.7 (23.7, 31.8)	27.7 (23.7, 31.9)	27.8 (23.8, 31.6)	0.806	0.014	11.2
Ethnicity, *n* (%)				0.119	0.124				0.949	0.006	0.0
White	1,226 (68.8)	1,118 (69.4)	108 (63.5)			319 (64.1)	213 (64.2)	106 (63.9)			
Others	556 (31.2)	494 (30.6)	62 (36.5)			179 (35.9)	119 (35.8)	60 (36.1)			
Comorbidities, *n* (%)											
Congestive heart failure	1,068 (59.9)	965 (59.9)	103 (60.6)	0.854	0.015	299 (60.0)	200 (60.2)	99 (59.6)	0.928	0.009	0.0
Diabetes	786 (44.1)	729 (45.2)	57 (33.5)	0.003	0.241	174 (34.9)	117 (35.2)	57 (34.3)	0.847	0.019	0.0
Chronic renal disease	614 (34.5)	582 (36.1)	32 (18.8)	< 0.001	0.395	87 (17.5)	55 (16.6)	32 (19.3)	0.474	0.068	0.0
Cerebrovascular disease	266 (14.9)	237 (14.7)	29 (17.1)	0.412	0.065	82 (16.5)	55 (16.6)	27 (16.3)	0.980	0.002	0.0
Chronic pulmonary disease	591 (33.2)	535 (33.2)	56 (32.9)	0.948	0.005	166 (33.3)	111 (33.4)	55 (33.1)	0.964	0.004	0.0
Sepsis	1,145 (64.3)	1,008 (62.5)	137 (80.6)	< 0.001	0.409	396 (79.5)	263 (79.2)	133 (80.1)	0.821	0.022	0.0
Clinical scores											
GCS	14.0 (10.0, 15.0)	14.0 (10.0, 15.0)	12.0 (7.0, 14.0)	< 0.001	0.406	13.0 (7.0, 14.0)	13.0 (7.0, 15.0)	12.0 (8.0, 14.0)	0.164	0.058	0.0
SOFA	6.0 (3.0, 9.0)	6.0 (3.0, 9.0)	7.0 (4.0, 10.0)	< 0.001	0.321	7.0 (4.0, 10.0)	6.0 (4.0, 10.0)	7.0 (4.0, 10.0)	0.596	0.045	0.0
Vital sign											
Heart rate, beats/min	82.4 (72.6, 92.7)	82.2 (72.3, 92.5)	84.8 (75.0, 96.5)	0.036	0.191	84.2 (733, 95.6)	84.0 (71.5, 95.8)	84.4 (74.8, 94.8)	0.824	0.04	0.0
Respiratory rate, beats/min	19.1 (17.1, 21.7)	19.0 (17.1, 21.6)	19.8 (17.1, 22.6)	0.082	0.131	19.8 (17.4, 22.7)	19.8 (17.5, 22.6)	19.7 (17.1, 22.5)	0.551	0.032	0.0
SBP, mmHg	111.4 (103.3, 122.0)	111.7 (103.3, 122.2)	109.5 (1,044, 118.7)	0.425	0.051	109.9 (103.1, 119.9)	109.9 (102.2, 120.4)	1,098 (104.5, 119.1)	0.636	0.052	0.0
DBP, mmHg	58.2 (52.2, 65.6)	58.1 (52.1, 65.5)	59.7 (53.4, 68.6)	0.007	0.249	61.2 (54.5, 68.8)	61.4 (55.0, 69.3)	60.2 (53.5, 68.5)	0.377	0.05	0.0
MBP, mmHg	74.2 (68.1, 80.1)	74.2 (68.0, 80.1)	74.9 (68.3, 81.5)	0.184	0.163	75.6 (69.6, 82.2)	75.8 (70.0, 82.7)	74.9 (683, 81.6)	0.355	0.059	0.0
Temperature, °C	36.8 (36.5, 37.1)	36.8 (36.5, 37.1)	36.9 (36.6, 37.2)	< 0.001	0.171	36.8 (36.6, 37.2)	36.8 (36.5, 37.2)	36.9 (36.6, 37.2)	0.337	0.037	0.0
SpO_2_, %	97.4 (96.0, 98.7)	97.4 (96.0 98.7)	97.6 (96.0, 98.7)	0.475	0.060	97.7 (96.1, 98.8)	97.6 (96.1, 98.8)	97.7 (96.0, 98.7)	0.870	0.010	8.6
Urine output, ml	1562.5 (946.5, 2375.0)	1562.5 (950.0, 2368.8)	1552.5 (910.3, 2423.3)	0.896	0.009	1638.5 (993.5, 2348.8)	1643.5 (996.3, 2275.3)	1627.5 (932.5, 2586.0)	0.809	0.004	0.0
Laboratory test											
Hemoglobin, g/dl	10.6 (9.4, 12.2)	10.5 (9.4, 12.1)	11.1 (9.3, 13.0)	0.031	0.191	11.0 (9.7, 12.9)	11.0 (9.8, 12.9)	11.1 (9.3, 12.9)	0.416	0.017	5.4
Platelets, 10^9^/L	209.0 (157.5, 268.6)	210.5 (159.0, 271.0)	197.0 (137.9, 250.0)	0.011	0.150	202.5 (147.6, 254.0)	205.0 (153.4, 255.4)	200.0 (139.5, 251.0)	0.407	0.043	7.7
WBC, 10^9^/L	11.9 (9.1, 15.5)	11.8 (9.0, 15.3)	12.5 (9.5, 16.8)	0.079	0.089	12.4 (9.4, 16.2)	12.4 (9.4, 16.2)	12.4 (9.3, 16.2)	0.9	0.009	5.1
BUN, mg/dl	25.0 (17.0, 41.5)	25.5 (17.5, 42.0)	21.8 (15.0, 34.3)	0.003	0.175	21.8 (15.6, 34.5)	22.0 (16.1, 34.5)	21.3 (15.1, 34.0)	0.549	0.017	6.3
Calcium, mmol/L	8.4 (7.9, 8.9)	8.4 (8.0, 8.9)	8.2 (7.7, 8.7)	< 0.001	0.404	8.2 (7.8, 8.7)	8.2 (7.8, 8.7)	8.2 (7.7, 8.7)	0.923	0.045	3.7
Creatinine, mg/dl	1.2 (0.9, 2.0)	1.3 (0.9, 2.1)	1.1 (0.8, 1.9)	0.048	0.086	1.1 (0.9, 1.84)	1.1 (0.9, 1.84)	1.1 (0.8, 1.8)	0.499	0.083	0.0
Glucose, mg/dl	143.3 (117.0, 185.1)	142.8 (117.5, 187.0)	144.5 (113.9, 177.6)	0.515	0.102	137.8 (115.0, 175.5)	135.5 (115.0, 173.9)	144.8 (116.0, 178.8)	0.309	0.057	0.0
Sodium, mmol/L	138.0 (135.5, 140.5)	138.0 (135.5, 140.0)	138.0 (135.5, 141.0)	0.606	0.090	138.5 (136.0, 141.0)	138.5 (136.5, 140.9)	138.0 (135.6, 141.0)	0.270	0.029	3.2
Potassium, mmol/L	4.3 (3.9, 4.7)	4.3 (4.0, 4.7)	4.2 (3.8, 4.7)	0.092	0.15	4.2 (3.9, 4.6)	4.2 (3.9, 4.6)	4.2 (3.8, 4.7)	0.858	0.042	10.2
pH	7.38 (7.33, 7.43)	7.38 (7.34, 7.43)	7.38 (7.32, 7.43)	0.434	0.036	7.38 (7.33, 7.43)	7.38 (7.34, 7.43)	7.38 (7.32, 7.43)	0.434	0.092	4.1
Lactate, mmol/L	1.7 (1.3, 2.4)	1.7 (1.3, 2.4)	1.8 (1.3, 2.7)	0.045	0.168	1.8 (1.3, 2.6)	1.7 (1.3, 2.6)	1.8 (1.3, 2.5)	0.405	0.061	6.9
PT, seconds	14.3 (12.8, 16.4)	14.3 (12.8, 16.4)	14.4 (12.6, 16.4)	0.938	0.076	14.3 (12.9, 16.4)	14.3 (12.9, 16.5)	14.3 (12.6, 16.4)	0.554	0.014	0.0
PPT, seconds	41.8 (31.2, 62.5)	41.7 (31.1, 63.2)	42.0 (32.8, 58.5)	0.949	0.064	41.5 (31.1, 61.2)	41.4 (30.5, 64.0)	41.7 (32.8, 57.6)	0.716	0.053	0.0
Clinical Therapy, *n* (%)											
Vasopressor	726 (40.7)	652 (40.4)	74 (43.5)	0.437	0.062	225 (45.2)	154 (46.4)	71 (42.8)	0.429	0.076	0.0
RRT	180 (10.1)	154 (9.6)	26 (15.3)	0.018	0.175	60 (12.0)	36 (10.8)	24 (14.5)	0.226	0.084	0.0
Mechanical ventilation	1706 (95.7)	1,541 (95.6)	165 (97.1)	0.369	0.078	481 (96.6)	320 (96.4)	161 (97.0)	0.666	0.042	0.0

BMI, body mass index; BUN, blood urea nitrogen; DBP, diastolic blood pressure; MBP, mean blood pressure; GCS, Glasgow Coma Scale; pH, hydrogen ion concentration; PSM, propensity-score matching; PT, prothrombin time; RRT, renal replacement therapy; SBP, systolic blood pressure; SOFA, sequential organ failure assessment; SMD, standardized mean difference; SpO2, oxygen saturation; WBC, white blood cell.

Patients in the thiamine group seemed to be younger compared with non-thiamine group; had a greater proportion of male, a lower GCS score, and a higher SOFA score. Moreover, they had reduced rates of diabetes and chronic renal disease but a greater incidence of sepsis. They also had higher values of heart rate, diastolic blood pressure, temperature, hemoglobin, and lactate, and decreased levels of platelets, creatinine, BUN, and calcium. Moreover, the thiamine group’s patients more likely required RRT.

Based on an adjusted PSM, there was no difference in baseline traits among the two groups when 332 cases who did not receive thiamine treatment were contrasted with 166 patients who did. The quality of the matched samples was evaluated by graphing the propensity scores of the two groups (Supplementary Figure S1). In addition, the Standardized mean difference (SMD) was calculated for the thiamine and non-thiamine groups for the original and PSM cohorts. Our results showed that 1:2 PSM yielded smaller SMD than the original cohort for all baseline measures and SMD values less than 0.1.

### KM survival analysis

**Figure 2:**
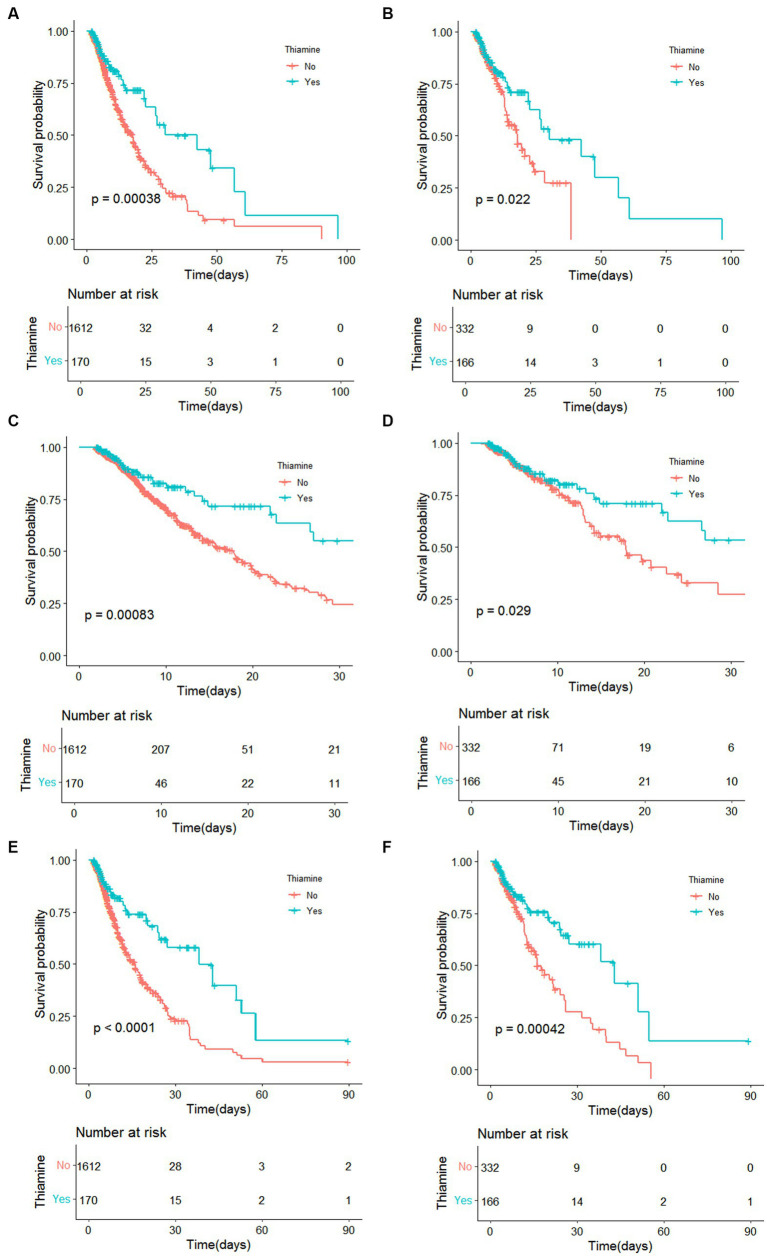
Kaplan–Meier survival curves between the thiamine and none-thiamine groups. **(A)** the original population of in-hospital mortality risk; **(B)** After propensity score matching adjustment of in-hospital mortality risk; **(C)** The original population of 30d ICU mortality risk; **(D)** After propensity score matching adjustment of 30d ICU mortality risk; **(E)** the original population of 90d ICU mortality risk; **(F)** After propensity score matching adjustment of 90d ICU mortality risk.

KM survival curves suggested that in-hospital, 30-day, and 90-day mortality differed significantly among the thiamine and non-thiamine groups (Figure 2). Figure 2A demonstrated that in the initial population, the thiamine group experienced a smaller in-hospital mortality rate compared with the non-thiamine group (*p* < 0.001). After PSM, the result of KM survival curve in the PSM population was in keep with that in the original population (*p* = 0.022, Figure 2B). Figure 2C demonstrated that in the initial population, the thiamine group possessed a smaller 30-day mortality rate than the non-thiamine group (*p* < 0.001), consistent with the results in the PSM population (*p* = 0.029, Figure 2D). Moreover, the 90-day mortality of the thiamine group was also lower compared with the non-thiamine group in the initial population (*p* < 0.001, Figure 2E) and the PSM population (*p* < 0.001, Figure 2F).

### Cox proportional-hazard regression models

The results of the multicollinearity diagnosis are shown in Supplementary Table S2. None of the VIFs exceeded 5 indicates that there was no multicollinearity among the variables. Cox proportional hazards models were further analyzed to reveal the relationship between thiamine supplementation and prognosis, and the results are reported in Table 2.

**Table 2:** Results of Cox proportional hazard models.

Category	Models	Original population		PSM population	
		HR (95% CI)	*p* value	HR (95% CI)	*p* value
In-hospital mortality	Crude model	0.507 (0.347–0.741)	< 0.001	0.586 (0.369–0.931)	0.024
	Model 1^a^	0.615 (0.414–0.912)	0.015	0.633 (0.395–0.948)	0.046
	Model 2^b^	0.615 (0.433–0.965)	0.03	0.627 (0.391–0.918)	0.042
	Model 3^c^	0.611 (0.406–0.918)	0.017	0.634 (0.394–0.929)	0.041
	Model 4^d^	0.554 (0.368–0.833)	0.004	0.539 (0.331–0.877)	0.012
	Model 5^e^	0.579 (0.381–0.881)	0.010	0.546 (0.327–0.912)	0.020
	Model 6^f^	0.605 (0.397–0.921)	0.019	0.559 (0.334–0.935)	0.027
30-d mortality	Crude model	0.506 (0.337–0.760)	< 0.001	0.597 (0.374–0.952)	0.030
	Model 1^a^	0.596 (0.391–0.909)	0.016	0.644 (0.401–0.931)	0.028
	Model 2^b^	0.631 (0.412–0.968)	0.034	0.635 (0.394–0.950)	0.041
	Model 3^c^	0.617 (0.401–0.949)	0.028	0.649 (0.402–0.920)	0.037
	Model 4^d^	0.562 (0.364–0.865)	0.008	0.566 (0.347–0.923)	0.022
	Model 5^e^	0.597 (0.385–0.925)	0.021	0.562 (0.337–0.938)	0.027
	Model 6^f^	0.618 (0.398–0.960)	0.030	0.586 (0.330–0.962)	0.035
90-d mortality	Crude model	0.517 (0.354–0.756)	< 0.001	0.586 (0.369–0.931)	0.023
	Model 1^a^	0.631 (0.425–0.937)	0.022	0.633 (0.395–0.923)	0.036
	Model 2^b^	0.665 (0.445–0.993)	0.046	0.627 (0.391–0.945)	0.032
	Model 3^c^	0.631 (0.419–0.948)	0.026	0.634 (0.394–0.921)	0.030
	Model 4^d^	0.573 (0.381–0.862)	0.007	0.539 (0.331–0.877)	0.012
	Model 5^e^	0.603 (0.397–0.916)	0.017	0.546 (0.327–0.912)	0.020
	Model 6^f^	0.626 (0.411–0.953)	0.029	0.559 (0.334–0.935)	0.027

HR, hazard ratio; CI, confidence interval; PSM, propensity-score matching. ^a^Model 1 was adjusted for demographic features, including age, gender, ethnicity, and BMI. ^b^Model 2 was additionally adjusted for comorbidities, including congestive heart failure, chronic renal disease, cerebrovascular disease, chronic pulmonary disease, and sepsis. ^c^Model 3 was additionally adjusted for clinical scores, including GCS, SOFA. ^d^Model 4 was additionally adjusted for vital signs, including heart rate, respiratory rate, SBP, DBP, MBP, temperature, SpO2, and urine output. ^e^Model 5 was additionally adjusted for laboratory tests, including hemoglobin, platelets, white blood cell, BUN, calcium, creatinine, glucose, sodium, potassium, lactate, PT, and PTT; f Model 6 was additionally adjusted for clinical therapy, including renal replacement therapy, mechanical ventilation, and vasopressor.

Thiamine administration was substantially linked with a 49% decrease in the probability of in-hospital mortality in the original group, according to a crude model of univariate Cox regression analysis (HR: 0.507, 95% CI: 0.347–0.741, *p* < 0.001). In the completely adjusted model, multivariate analyses revealed a notable beneficial impact of thiamine supplementation on in-hospital mortality behind adjusting for demographic characteristics, complications, clinical scores, vital signs, laboratory parameters, and clinical interventions (*p* = 0.019). Following PSM, the crude models discovered that thiamine treatment was connected to a 41% decrease among the risk of in-hospital mortality (*p* = 0.024). Likewise, the completely adjusted model based on the PSM population, which is adjusted for a series of confounds, also showed a similar result (HR: 0.559, 95% CI: 0.334–0.935, *p* = 0.027).

With regard to the 30-d mortality, a decreased mortality risk in the original population was observed in the crude (HR: 0.506, 95% CI: 0.337–0.760, *p* < 0.001) and completely adjusted models (HR: 0.618, 95% CI: 0.398–0.960, *p* = 0.032). After PSM, a similar trend was also found in the crude (HR: 0.597, 95% CI: 0.374–0.952, *p* = 0.030) and completely adjusted models (HR: 0.586, 95% CI: 0.330–0.962, *p* = 0.035).

Concerning the 90-d mortality, the crude model demonstrated a significant survival benefit of thiamine supplement in the original (HR: 0.517, 95% CI: 0.354–0.756, *p* < 0.001) and PSM populations (HR: 0.586, 95% CI: 0.369–0.931, *p* = 0.024). After adjustments of various confounders, an important positive impact of thiamine treatment was also observed in the original (HR: 0.626, 95% CI: 0.411–0.953, *p* = 0.029) and PSM populations (HR: 0.559, 95% CI: 0.334–0.935, *p* = 0.027).

According to the Schoenfeld residual plots (Supplementary Figure S2) and the deviance residual plots (Supplementary Figure S3), the formulated Cox regression models conformed to the PH hypothesis, indicating that the HR estimations were valid.

### Subgroup analysis

**Figure 3:**
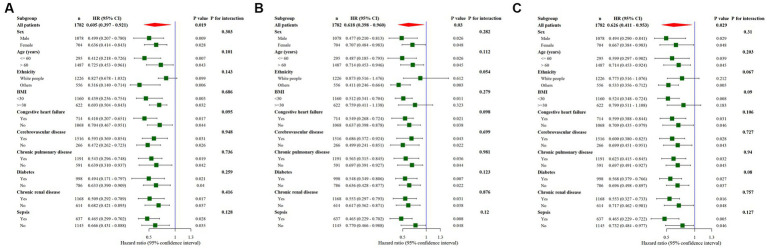
Subgroup analysis of the association between thiamine use and outcomes in critically ill patients with myocardial infarction. **(A)** The subgroup analysis of in-hospital mortality risk. **(B)** The subgroup analysis of 30d mortality risk. **(C)** The subgroup analysis of 90d mortality risk. HR, hazard ratio; CI, confidence interval.

Subgroup analysis was performed for in-hospital, 30-d, and 90-d mortality (Figure 3). Results showed that thiamine use contributed to a survival beneficial in almost all subgroups. Furthermore, there was no discernible interaction, in all strata, when comparing the thiamine and none-thiamine groups.

### Sensitivity analysis

After excluding individuals with missing data, a total of 1,426 patients were included in the sensitivity analysis with 139 (9.7%) and 1,287 (90.2%) cases in the thiamine and non-thiamine groups, respectively. The results of sensitivity analyses showed that thiamine usage had a significant survival benefit among critically ill patients with MI, indicating that our results were robust and reliable (Supplementary Table S3; Supplementary Figure S4).

## Discussion

Our study was the first to evaluate the effects of thiamine supplementation on the outcome of critically ill patients with MI from the MIMIC-IV database. Results revealed a strong association between thiamine supplementation and reduced risk of ICU, 30-d, and 90-d mortality in MI patients regardless of adjustments for multifarious confounding factors using the Cox regression models. After verification by PSM and subgroup analysis, the results also supported that thiamine supplementation contributed to a survival benefit in critically ill patients with MI.

Thiamine, also known as vitamin B1, was an essential micronutrient and co-factor involved in the important metabolism of the body. Thiamine is essential for the Krebs cycle’s oxidative decarboxylation, which takes place in the mitochondria, for the creation of ATP and offers energy for cells. Hence, thiamine deficiency may be limit mitochondrial function and reduce ATP production, leading to severe cardiovascular, metabolic, neurological, respiratory, gastrointestinal, and musculoskeletal system disorders (12, 23). Conversely, thiamine supplementation may improve patient outcomes by restoring mitochondrial function and perfusion in harmed tissues, which will lessen organ dysfunction. Moreover, thiamine defends human infragenicular artery smooth muscle cells from glucose- and insulin-mediated proliferation, which are known to be essential for the formation of the atherosclerotic plaque; thus, thiamine supplementation results in improved cardiac functions and hemodynamic features, as well as decreased in systemic vascular resistance. Thiamine also counteracts the damaging effects of high glucose concentrations on endothelial cells by reducing intracellular protein glycosylation (24). Additionally, thiamine is an antioxidant involved in many redox reactions. Critically ill patients are prone to inflammatory responses and tissue hypoxia, which disrupts the balance of oxidative and antioxidant systems within the body and increases the products of oxidative stress, such as reactive oxygen species (25). Based on the inhibition of lipid peroxidation and oleic acid oxidation mechanisms, thiamine supplementation could improve the oxidative stress status (26). Thiamine supplementation also has a promoting effect on the body’s immune system and immune cells. For example, it protects macrophages from oxidative stress and has an antioxidant impact on neutrophil cells. Then, by preventing P43’s intracellular function, it also further helps the p53 inhibitory protein work as an anti-inflammatory, which is considered as the pathophysiological basis of thiamine in improving poor outcomes among critically ill patients (27).

A large number of evidences shows that 20% critically ill patients often have thiamine deficiency during hospitalization, which may be due to metabolic stress, reduced or poor nutritional intake, and multiple comorbidities (28, 29). Under-nutrition status is common among critically ill patients and may result in inadequate thiamine intake. Notably, thiamine deficiency is difficult to promptly detect in critically ill patients due to the symptomatic lack of sensitivity and specificity. This defect can also lead to peripheral neuropathy, congestive heart failure, gastrointestinal beriberi, Korsarkov’s syndrome, and Wernicke’s encephalopathy, as well as accelerate the development of complications, such as confusion, unexplained lactic acidosis, and gastrointestinal dysfunction (18). Cardiomyocytes require a sustained energy supply, and abnormalities of the aerobic respiratory pathways caused by thiamine deficiency interrupt normal cardiac function owing to the endovascular dysfunction caused by the thiamine-dependent nitric oxide synthase. Thiamine deficiency is also reportedly associated significantly with CVD such as heart failure, MI, and conduction block, as well as its risk factors (obesity and diabetes) (12). Furthermore, endothelial dysfunction and chronic vascular inflammation are prominent risk factors for the development of atherosclerosis and CVD (23, 30). Thiamine deficiency may aggravate endothelial dysfunction and chronic vascular inflammation, resulting in the loss of arterial vascular resistance that eventually develop into CVD. Therefore, thiamine supplementation may provide an unexpected benefit to the prognosis and outcome in patients with CVD.

Thiamine is transported primarily through red blood cells into organs with high energy and metabolic needs, resulting in the heart being the first to be affected when thiamine deficiency occurs. Moreover, thiamine deficiency interferes with how the regular CV system functions because of the elevated lactate level caused by the accumulation of pyruvate. These lead to raising ventricular filling pressure and oxygen demand (12). Numerous studies have shown how thiamine supplementation can prevent CVD. A meta-analysis summarizing data from two randomized controlled trials has shown that thiamine supplementation induces an overall improvement in left ventricular ejection fraction (LVEF) by 3.28% among patients with systolic heart failure (31). Yang et al. indicated that the probability of in-hospital death in patients with heart failure is dramatically reduced by 26% when thiamine supplementation is used (8). Additionally, Schoenenberger et al. demonstrated an improvement in LVEF among patients with heart failure who have received 300 mg/day thiamine supplementation for 4 weeks (32). Although existing evidence suggests that thiamine supplementation may contribute to increased therapeutic effect of CVD, the recommended reference intake and methods of intake are unclear. The lack of consistent results in clinical trials may explain the low proportion of thiamine supplementation in MI patients in this study.

Similar results have been found in other studies of critically ill patients, indicating that thiamine supplementation may be beneficial for improving outcomes in critically ill patients. For instance, patients with ventilator-associated pneumonia given thiamine supplementation have a significantly decreasing ICU and in-hospital mortality when comparing those untreated with thiamine (10). Another retrospective study has demonstrated that HAT therapy including thiamine supplementation could reduce in-hospital mortality in patients with sepsis (29). Taking the results of this study together with the current evidence, a simple, practical, and risk-free technique to enhance cardiac function is thiamine supplementation. It is worthy of being recommended to enhance survival outcomes in critically ill patients with MI.

To the best of our understanding, this research effort was the first to investigate the relevance between outcomes in MI patients and thiamine and had several strengths. First, our results were validated by PSM and an array of Cox regression models adjusting for various confounders, and the same results were achieved. Second, the MIMIC-IV database embraced quite a few of patient populations that served as a strong foundation for our study. However, we must acknowledge several limitations. First, single-center retrospective observational studies cannot avoid selection bias. Second, we were unable to determine whether thiamine benefited all MI patients or only thiamine-deficient individuals because MIMIC-IV lacked baseline thiamine levels. Third, this work did not consider the dose and duration of thiamine supplementation. Fourth, studies using MI to fill in the data may deviate from the true value. Therefore, more well-planned clinical trials are required to investigate the prognostic relationship between thiamine and MI in the future.

## Conclusion

Thiamine supplementation contributes to a potential survival benefit in critically ill patients with MI. Considering that thiamine is convenient, safe, and low cost, it has an excellent application prospect in critically ill patients. However, the effect of thiamine supplementation requires further multicenter and well-designed clinical trials to provide more convincing evidence and validate the findings.

## Data availability statement

Publicly available datasets were analyzed in this study. This data can be found at: https://mimic.physionet.org/.

## Ethics statement

Ethical approval was not required for the study involving humans in accordance with the local legislation and institutional requirements. Written informed consent to participate in this study was not required from the participants or the participants’ legal guardians/next of kin in accordance with the national legislation and the institutional requirements.

## Author contributions

JWu, SY, and JWa conceived of and designed the work. SY, YZ, and JWa acquired and check the data. EY, XL, and YH performed statistical and computational analyses. SY, YH, and DN assisted the analysis and explain of statistical methods. JWa, JH, XH, and EY provided professional clinical analyses. SY, JWa, JyW, and YZ drafted the work. All authors read and approved the final manuscript. All authors contributed to the article and approved the submitted version.

## Funding

This study was supported by the Competitive Project of Financial Special Funds for Science and Technology of Zhanjiang City (2022A01161) and the Affiliated Hospital of Guangdong Medical University Clinical Research Program (LCYJ2019B005).

## Conflict of interest

The authors declare that the research was conducted in the absence of any commercial or financial relationships that could be construed as a potential conflict of interest.

## Publisher’s note

All claims expressed in this article are solely those of the authors and do not necessarily represent those of their affiliated organizations, or those of the publisher, the editors and the reviewers. Any product that may be evaluated in this article, or claim that may be made by its manufacturer, is not guaranteed or endorsed by the publisher.
