# Supplementary material for: Thiamine administration may increase survival benefit in critically ill patients with myocardial infarction
Source: Front Nutr. 2023 Aug 29;10:1227974. doi: 10.3389/fnut.2023.1227974 (PMC10497214; doi:10.3389/fnut.2023.1227974)
Supplement: Supplementary file 1 [file Table_1.docx]

**Supplementary Table 1. *P* values for the normality tests**

| Variables | Original population  (n = 1782) | PSM population  (n = 498) |
| --- | --- | --- |
| BMI, kg/m^2^ | < 0.001 | < 0.001 |
| Age, years | < 0.001 | 0.007 |
| Hemoglobin, g/dl | < 0.001 | < 0.001 |
| Platelets, 10^9^/L | < 0.001 | < 0.001 |
| WBC, 10^9^/L | < 0.001 | < 0.001 |
| BUN, mg/dl | < 0.001 | < 0.001 |
| Calcium, mmol/L | < 0.001 | 0.003 |
| Creatinine, mg/dl | < 0.001 | < 0.001 |
| Glucose, mg/dl | < 0.001 | < 0.001 |
| Sodium, mmol/L | < 0.001 | < 0.001 |
| Potassium, mmol/L | < 0.001 | < 0.001 |
| PT, seconds | < 0.001 | < 0.001 |
| PPT, seconds | < 0.001 | < 0.001 |
| Heart rate, beats/min | < 0.001 | 0.002 |
| SBP, mmHg | < 0.001 | < 0.001 |
| DBP, mmHg | < 0.001 | < 0.001 |
| MBP, mmHg | < 0.001 | < 0.001 |
| Respiratory rate, beats/min | < 0.001 | 0.004 |
| Temperature, ℃ | < 0.001 | < 0.001 |
| SpO^2^, % | < 0.001 | < 0.001 |
| Lactate, mmol/L | < 0.001 | < 0.001 |
| pH | < 0.001 | 0.001 |
| Urine output, ml | < 0.001 | < 0.001 |
| SOFA | < 0.001 | < 0.001 |
| GCS | < 0.001 | < 0.001 |
