# Supplementary material for: Thiamine administration may increase survival benefit in critically ill patients with myocardial infarction
Source: Front Nutr. 2023 Aug 29;10:1227974. doi: 10.3389/fnut.2023.1227974 (PMC10497214; doi:10.3389/fnut.2023.1227974)
Supplement: Supplementary file 2 [file Table_2.docx]

**Supplementary Table 2. Collinearity diagnostics**

| **Variables** | **VIF** |
| --- | --- |
| Thiamine | 1.093 |
| Age | 1.499 |
| Gender | 1.129 |
| Ethnicity | 1.066 |
| BMI | 1.144 |
| Congestive heart failure | 1.205 |
| Diabetes | 1.440 |
| Peripheral vascular disease | 1.191 |
| Cerebrovascular disease | 1.083 |
| Chronic pulmonary disease | 1.087 |
| Chronic renal disease | 1.607 |
| Sepsis | 1.405 |
| GCS | 1.993 |
| SOFA | 3.992 |
| Hemoglobin | 1.516 |
| Platelets | 1.204 |
| WBC | 1.204 |
| BUN | 2.208 |
| Calcium | 1.199 |
| Creatinine | 2.207 |
| Glucose | 1.460 |
| Sodium | 1.128 |
| Potassium | 1.356 |
| PT | 1.109 |
| PPT | 1.115 |
| Lactate | 1.531 |
| PH | 2.160 |
| SpO2 | 1.208 |
| Heart rate | 1.460 |
| SBP | 2.867 |
| DBP | 4.253 |
| MBP | 4.112 |
| Respiratory rate | 1.416 |
| Temperature | 1.203 |
| Urine output | 1.293 |
| RRT | 1.526 |
| Vasopressor | 1.757 |
| Mechanical ventilation | 1.088 |

BMI, body mass index; BUN, blood urea nitrogen; DBP, diastolic blood pressure; MBP, mean blood pressure; GCS, Glasgow Coma Scale; pH, hydrogen ion concentration; PT, prothrombin time; RRT, renal replacement therapy; SBP, systolic blood pressure; SOFA, sequential organ failure assessment; SpO2, oxygen saturation; WBC, white blood cell.
