# Supplementary material for: Thiamine administration may increase survival benefit in critically ill patients with myocardial infarction
Source: Front Nutr. 2023 Aug 29;10:1227974. doi: 10.3389/fnut.2023.1227974 (PMC10497214; doi:10.3389/fnut.2023.1227974)
Supplement: Supplementary file 3 [file Table_3.docx]

**Supplementary Table 3. Results of sensitivity analyses by Cox proportional hazard models**

| **Category** | **Models** | **Original population** | | **PSM population** | |
| --- | --- | --- | --- | --- | --- |
|  |  | **HR (95% CI)** | ***P* value** | **HR (95% CI)** | ***P* value** |
| In-hospital mortality | Crude model | 0.485 (0.320-0.733) | < 0.001 | 0.602 (0.362-0.994) | 0.042 |
|  | Model 1^a^ | 0.596 (0.388-0.915) | 0.018 | 0.613 (0.385-0.918) | 0.039 |
|  | Model 2^b^ | 0.609 (0.392-0.946) | 0.027 | 0.597 (0.361-0.931) | 0.012 |
|  | Model 3^c^ | 0.574 (0.366-0.899) | 0.015 | 0.584 (0.334-0.871) | 0.031 |
|  | Model 4^d^ | 0.481 (0.299-0.772) | 0.002 | 0.589 (0.311-0.897) | 0.022 |
|  | Model 5^e^ | 0.513 (0.312-0.843) | 0.008 | 0.566 (0.387-0.952) | 0.031 |
|  | Model 6^f^ | 0.586 (0.353-0.974) | 0.039 | 0.394 (0.192-0.805) | 0.011 |
| 30-d mortality | Crude model | 0.366 (0.222-0.602) | < 0.001 | 0.464 (0.267-0.808) | 0.007 |
|  | Model 1^a^ | 0.481 (0.289-0.800) | 0.004 | 0.510 (0.284-0.918) | 0.025 |
|  | Model 2^b^ | 0.489 (0.292-0.819) | 0.006 | 0.478 (0.259-0.879) | 0.018 |
|  | Model 3^c^ | 0.532 (0.315-0.897) | 0.017 | 0.587 (0.259-0.925) | 0.027 |
|  | Model 4^d^ | 0.490 (0.288-0.835) | 0.009 | 0.591 (0.387-0.893) | 0.033 |
|  | Model 5^e^ | 0.465 (0.367-0.828) | 0.011 | 0.408 (0.322-0.848) | 0.029 |
|  | Model 6^f^ | 0.488 (0.280-0.849) | 0.012 | 0.365 (0.172-0.777) | 0.009 |
| 90-d mortality | Crude model | 0.485 (0.320-0.733) | < 0.001 | 0.527 (0.318-0.875) | 0.013 |
|  | Model 1^a^ | 0.621 (0.406-0.949) | 0.022 | 0.593 (0.380-0.874) | 0.019 |
|  | Model 2^b^ | 0.635 (0.412-0.979) | 0.039 | 0.583 (0.368-0.872) | 0.018 |
|  | Model 3^c^ | 0.588 (0.370-0.932) | 0.024 | 0.614 (0.438-0.879) | 0.029 |
|  | Model 4^d^ | 0.572 (0.358-0.916) | 0.020 | 0.579 (0.401-0.827) | 0.022 |
|  | Model 5^e^ | 0.663 (0.337-0.976) | 0.014 | 0.561 (0.344-0.871) | 0.017 |
|  | Model 6^f^ | 0.505 (0.303-0.841) | 0.009 | 0.352 (0.173-0.716) | 0.004 |

HR, hazard ratio; CI, confidence interval; PSM, propensity-score matching.

a Model 1 was adjusted for demographic features, including age, gender, ethnicity, and BMI; b Model 2 was additionally adjusted for comorbidities, including congestive heart failure, chronic renal disease, cerebrovascular disease, chronic pulmonary disease, and sepsis; c Model 3 was additionally adjusted for clinical scores, including GCS, SOFA; d Model 4 was additionally adjusted for vital signs, including heart rate, respiratory rate, SBP, DBP, MBP, temperature, SpO2, and urine output; e Model 5 was additionally adjusted for laboratory tests, including hemoglobin, platelets, white blood cell, BUN, calcium, creatinine, glucose, sodium, potassium, lactate, PT, and PTT; f Model 6 was additionally adjusted for clinical therapy, including renal replacement therapy, mechanical ventilation, and vasopressor.
