# Supplementary figures and images for: Thiamine administration may increase survival benefit in critically ill patients with myocardial infarction
Source: Front Nutr. 2023 Aug 29;10:1227974. doi: 10.3389/fnut.2023.1227974 (PMC10497214; doi:10.3389/fnut.2023.1227974)

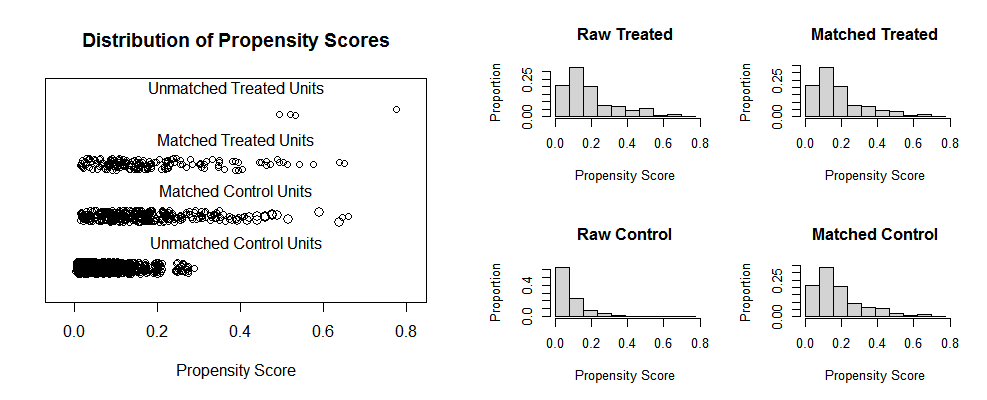

Supplement: Supplementary file 4 [file Image_1.TIF]

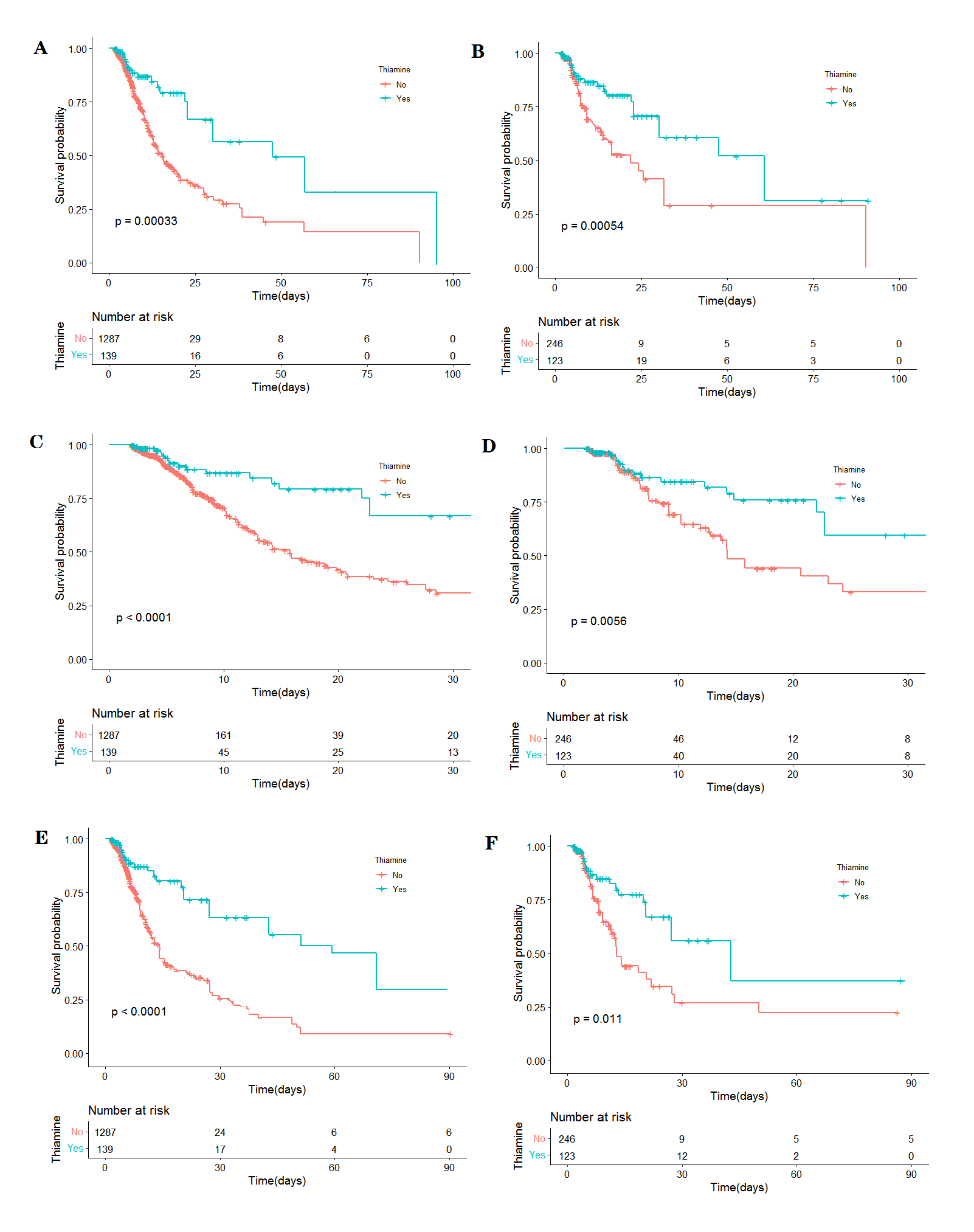

Supplement: Supplementary file 7 [file Image_4.TIF]
